# Optimizing glucocorticoid therapy in congenital adrenal hyperplasia and analog conditions: the intersection of dose reduction, patient care, and coverage in the US

**DOI:** 10.3389/fendo.2025.1603701

**Published:** 2025-09-09

**Authors:** Ahmed Khattab, Hyunwoo Kim, Mary Mulrooney, Brian Leinwand, Nandini Hadker, Samantha Cicero, Henry Cheng

**Affiliations:** ^1^ Rutgers University, New Brunswick, NJ, United States; ^2^ Neurocrine Biosciences, San Diego, CA, United States; ^3^ Trinity Life Sciences, Waltham, MA, United States

**Keywords:** glucocorticoid-reducing therapies, payer, congenital adrenal hyperplasia, survey, corticosteroids, glucocorticoid dose reduction

## Abstract

**Background:**

Glucocorticoid (GC) therapies treat many chronic conditions such as congenital adrenal hyperplasia (CAH), but long-term use carries risks of side effects (e.g., skeletal, cardiometabolic, mental health issues) that can negatively impact clinical and economic outcomes. Consequently, patients and providers seek to balance the lowest efficacious dose and side effect risk. To our knowledge, no research has analyzed US payer coverage decisions on medications that reduce GC reliance.

**Objective:**

To understand the significance and implications of US payer perceptions and coverage/access decisions for therapies reducing GC doses, which may be of relevance to new therapies for CAH.

**Methods:**

A literature review was paired with primary market research to identify and characterize 5 GC-reducing therapies to evaluate payer coverage policies. No identified therapies were currently approved or studied in CAH. Qualitative interviews (n=13) were also conducted across managed care organizations, pharmacy benefit managers, and managed Medicaid payers to supplement publicly available information.

**Results:**

GC-reducing therapies were desirable and therapeutically beneficial from payer perspectives based on market research of payer coverage policies; all therapies were covered in place of or in addition to GCs. Despite premium pricing vs. low-cost alternatives, all therapies evaluated were covered by some or all payers with prior authorization to label indication or trial criteria. Qualitative interviews revealed that payers clearly understood the clinical burden of long-term GC use; however, the economic burden was less understood. Payers stated that GC reduction is a secondary decision-making driver due to the focus on trial primary endpoints, contracting dynamics, lack of competitors, and small trial sample sizes. A subset of payers was interested in GC reduction data as a primary endpoint for rare diseases without treatment alternatives and in pediatric populations.

**Conclusions:**

Despite the premium price over GCs, GC-reducing therapies were covered in place of or in addition to GCs. Payers acknowledged the clinical value of reducing long-term GC use. Understanding what payers perceive as important criteria for coverage of GC-reducing medication may aid clinicians in evaluating utilization management criteria, such as step therapy, and increase access to medications aiming to reduce the patient burden associated with long-term GC use. This is particularly important in CAH where there is a high unmet need due to lifelong exposure to supraphysiologic doses of GCs.

## Highlights

Steroids are common treatments used in many diseases for different benefits such as to decrease inflammation or to replace hormones when a person’s body may not make enough. However, their use may cause side effects that can last a long time. The way insurance companies think about medications that reduce steroid dose, and how their decisions impact whether to cover a medication that reduces steroid dose, is not well understood. This study shows that insurance companies are concerned about side effects of steroids. Medications that reduced steroid dose were covered along with steroids or offered in place of steroids.

## Introduction

Glucocorticoid (GC) therapies are synthetic analogs of natural steroid hormones produced by the adrenal cortex that can be given systemically as a treatment for many diseases (1). While GC therapy is critical in the treatment of numerous inflammatory and immunologic conditions, chronic GC use carries significant risks of long-term comorbidities, including osteoporosis, adrenal suppression, metabolic disorders, immunosuppression, neuropsychiatric effects, and cardiovascular disease (1, 2). The use of long-term GCs and GC-related comorbidities have the potential to cause a substantial economic burden for patients and payers.

Although the economic burden of GC use has not been well studied, recent evidence suggests the economic cost is considerable, particularly in patients who take higher GC doses (3–5). Indeed, in a systematic literature review of disease states with long-term GC exposure (e.g., autoimmune diseases, asthma, lung diseases) healthcare costs increased correspondingly with GC dose (4). Costs ranged from approximately $5,700 in low-dose GC users (<7.5 mg prednisone or equivalent [30 mg hydrocortisone equivalents (HCe)]/day) to $29,000 in high-dose GC users (>15 mg prednisone or equivalent [60 mg HCe]/day) in per-annum incremental costs relative to non-users (4). Furthermore, in systemic lupus erythematosus (SLE), low-dose GC users (<7.5 mg prednisone [30 mg HCe] or equivalent/day) had mean incremental healthcare costs of $21,869 vs. $45,360 in high-dose GC users (>15 mg prednisone or equivalent [60 mg HCe]/day) (3). GC-related adverse events can also be particularly costly; for example in a systematic literature review of 47 studies assessing adverse events with GCs, 1-year per-patient costs were highest for stroke ($36,390 [2024 US dollars]), non-fatal myocardial infarction ($39,470), and fractures ($27,372) (5). Additionally, the use of GCs has been associated with reductions in health-related quality of life, which increases in magnitude as patients fill more GC prescriptions (6).

Congenital adrenal hyperplasia is a group of autosomal recessive disorders associated with impaired cortisol synthesis and includes classic and non-classic forms. Classic congenital adrenal hyperplasia, herein referred to as CAH, relies on long-term use of GCs. The most common cause of CAH is mutations in the *CYP21A2* gene that leads to 21-hydroxylase deficiency, which is associated with hyperandrogenism and variable degrees of cortisol and aldosterone deficiency (7–9). The classical form of CAH is typically diagnosed through prenatal or postnatal screening techniques, with an incidence of approximately 1 in 15,000 to 1 in 17,000 live births, which defines CAH as a rare disease under the Orphan Drug Act (10); the serious manifestations of the condition start at birth and require lifelong GC therapy with doses above physiologic levels, which can lead to long-term health challenges that can impact cardiometabolic risk, bone health, and quality of life, among other health domains (11, 12). CAH management involves a delicate balance between the risks associated with hyperandrogenism and those associated with chronic GC overexposure (11, 12). The variability in response to GCs in patients with CAH may lead to iatrogenic Cushing syndrome or adrenal crisis, which makes it challenging for clinicians and patients to manage the disease, ensure treatment adherence, and control adverse impacts of androgen excess (11, 12).

Since GCs have historically been the only medications used in CAH to manage an excess of adrenocorticotropic hormone (ACTH) and adrenal androgens, as well as replace cortisol, there is considerable unmet need in this patient population, particularly for patients who have burdensome GC dose-related adverse effects (13). However, novel treatments that reduce GC dose in other disease states where GCs are commonly prescribed, such as belimumab for SLE and sarilumab for polymyalgia rheumatics (PMR), have emerged (14–20). While most of these branded therapies have evidence to support that they can lower the required GC doses, they often also have utilization management criteria (e.g., prior authorization, step therapy) required for use (21).

To our knowledge, no research has been conducted to characterize United States (US) payer coverage decisions on GC-reducing medications and at the time of the analysis, no GC-reducing therapies were available for the treatment of CAH. To aid the optimization of treatment in patients with CAH, the objective of this study was to understand how payers perceive GC-reducing therapies in other disease states and make decisions related to their coverage and access as a proxy for future GC-reducing therapies in CAH.

## Methods

### Study design

A 2-phase approach was used in this study. First, a review of literature and market research were used to evaluate GC-reducing therapies and their payer coverage policies. Next, a cross-sectional study was conducted to understand health plans’ stated perspectives on the value of GC-reducing therapies, and to better characterize perspectives derived from their management actions (e.g., coverage criteria) of these therapies.

### Identification of therapies

**Figure 1:**
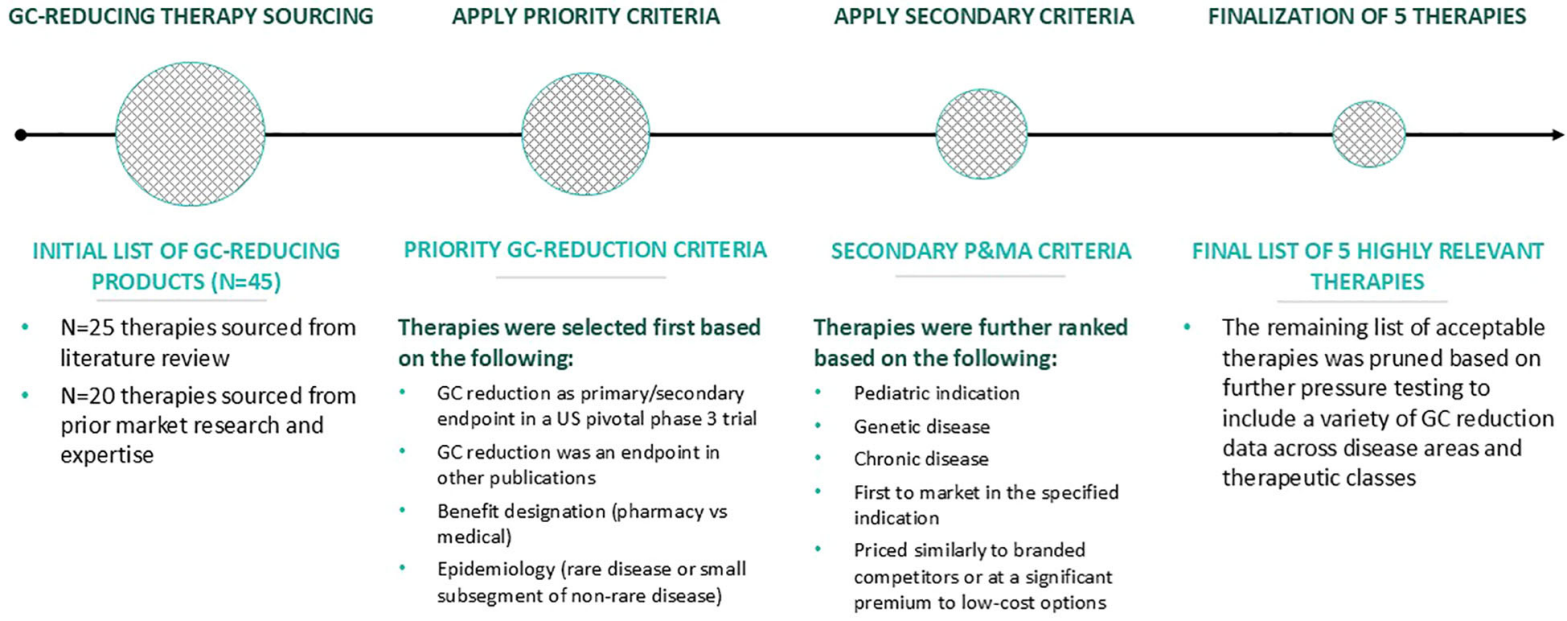
Approach to identifying GC-reducing therapies for evaluation. GC, glucocorticoid; P&MA, pricing and market access.

GC-reducing therapies were identified and analyzed (**Figure 1**). First, approximately 45 potential therapies that allow for a reduction in GC dose/utilization were identified from prior assessments, including a review of the literature and primary market research. Indications in which very high levels of GCs are typically used (>40 mg/m^2^/day HCe) were excluded to align with similar GC doses (< 40 mg/m^2^/day HCe) used in CAH. The therapies that had indications for which GCs were the primary treatment until specialized products became covered were then analyzed. As noted, at the time of analysis, no GC-reducing therapies were available for the treatment of CAH and, thus, none of the identified GC-reducing therapies were currently approved or studied in CAH. The therapies were selected based on the following criteria:

- GC reduction as a primary or secondary endpoint in one or more pivotal Phase 3 clinical trials in the US or as an endpoint in an observational study or other publication (non-clinical trial).
- Epidemiology of the therapy’s indication represented either a rare disease (defined as having a prevalence of less than 200,000 patients in the US) or a subset of a non-rare disease.
- Covered under US commercial 2023 health plans designated as pharmacy or medical.

Therapies were subsequently ranked in order of those meeting the highest to lowest number of the following preferred criteria:

- First therapy to receive Food and Drug Administration (FDA) approval in its specified treatment indication.
- Indicated to treat pediatric patients.
- Indicated in a genetic disease.
- Indicated in a chronic disease.
- Priced similarly to branded competitors or priced at a substantial premium to lower-cost options.

Based on this assessment and ranking, 5 therapies that included a variety of GC reduction data across diverse disease areas/therapeutic classes were analyzed in greater detail. Additional secondary research for the 5 selected therapies included indication, clinical evidence package, and payer policies (formulary status and prior authorization criteria) across 5 large national commercial plans (Aetna, Humana, Anthem, United HealthCare, and Cigna).

### Cross-sectional study (qualitative interviews)

Due to the limited information on the impact of GC reduction on multi-factorial formulary coverage decisions available through the literature review and market research, direct interviews were conducted with US decision-makers of national and regional managed care organizations (MCOs), pharmacy benefit managers (PBMs), and managed Medicaid health plans.

Payers were invited to participate through a healthcare research panel. Prior to participation, payers completed a self-administered, web-enabled screener to confirm eligibility to participate in the study. Payers who were eligible to participate must have met the following criteria:

- Voting member of a Pharmacy and Therapeutics Committee in their organization.
- At least 3 years and no more than 30 years of experience in their current position.
- Familiarity with at least 3 of the 5 selected therapy’s indication/therapeutic areas, with a self-assessed familiarity rating of at least 4 out of a possible 7 (scale 1 to 7, where 1 = least familiar and 7 = extremely familiar).
- Familiarity with their organization’s coverage policies for at least 3 of the 5 selected therapies in their specified indication/therapeutic areas, with a self-assessed familiarity rating of at least 4 out of a possible 7.

After the screening, eligible payers were invited to participate in a 45-minute in-depth telephone interview to capture their experiential background, familiarity with the selected GC-reducing therapies and relevant therapeutic areas, and insight regarding coverage and reimbursement decision-making. All interviews were conducted between December 2023 and January 2024.

#### Standard protocol approvals, registrations, and informed consent

This study was reviewed and determined to be exempt by ADVARRA, a central Institutional Review Board, and in compliance with ethical standards. All payers provided written consent via an online screener prior to participating in the qualitative interviews and received compensation for study participation.

#### Data analysis

Qualitative data derived from interviews were reviewed and assessed in aggregate and categorized according to key themes identified. Descriptive statistics were performed to describe the study population and quantitative responses captured during the interview, including responses captured on a 7-point Likert scale (1 = least familiar or knowledgeable and 7 = extremely familiar or knowledgeable). A higher score denoted more knowledge or familiarity with the topic of the survey question.

## Results

### Literature review and market research

#### Characteristics and coverage dynamics of selected GC-reducing therapies

Based on the assessment and ranking of GC-reducing therapies, the 5 therapies selected were adalimumab (Humira^®^), belimumab (Benlysta™), eteplirsen (Exondys 51^™^), rilonacept (Arcalyst^®^), and sarilumab (Kevzara^®^) (**Table 1**).

**Table 1:** Characteristics of selected payer-covered GC-reducing therapies evaluated.

Generic name (brand name)	Indication (population)^a^	RoA	GC reduction endpoint category	GC reduction data^b^	Rare indication	Pediatric indication^c^	Genetic disease	Chronic disease	First to market in indication	Priced similarly to branded competitors or substantial premium to low-cost options
Adalimumab (Humira)	Moderate to severe RA (adults)	SC	Retrospective study outcome	Yes	No	Yes	Yes	Yes	No	Yes
Belimumab (Benlysta)	SLE (adults)	IV/SC	Secondary	Yes	Yes	Yes	Yes	Yes	Yes	Yes
Eteplirsen (Exondys 51)	DMD (adults)	IV	Exploratory	No	Yes	Yes	Yes	Yes	Yes	Yes
Rilonacept (Arcalyst)	RP (aged ≥12 years)	SC	Secondary	Yes	Yes	No	Yes	Yes	Yes	Yes
Sarilumab (Kevzara)	PMR in GC non-responders or intolerance (adults)	SC	Secondary	Yes	Yes	No	Yes	Yes	Yes	Yes

**Table 1** summarizes the 5 GC-reducing therapies identified via a literature review and primary market research, their indications, RoA, how GC reduction was captured in studies, and whether each therapy met preferred criteria.

^a^Indication in which GCs were the treatment approach until the specialized product entered the market.

^b^Included data in a pivotal phase trial or as RWE.

^c^One of the FDA indications includes the pediatric population but may not have been the indication that was evaluated in the literature review.

DMD, Duchenne muscular dystrophy; GC, glucocorticoid; IV, intravenous; PMR, polymyalgia rheumatica; RA, rheumatoid arthritis; RoA, route of administration; RP, recurrent pericarditis; RWE, real-world evidence; SC, subcutaneous; SLE, systemic lupus erythematosus.

Pink boxes indicate characteristic is not present. Green boxes indicate characteristic is present.

All selected therapies had broad market access, were indicated in chronic diseases, and were in indications where the current treatment approach was GCs until a specialized product entered the market and had been covered throughout the years if it had successfully replaced or reduced GC use. The therapies included were purposefully diverse and had unique characteristics that made payer decision-making multi-factorial. Although adalimumab was not first to market in its specified indication or indicated in a rare disease, it was selected because it is the only approved rheumatoid arthritis (RA) therapy to demonstrate steroid reduction in a US-based trial and indicated in a subset of patients (those with moderate to severe RA) (22). Eteplirsen, indicated for Duchenne muscular dystrophy (DMD), was selected, partly due to it being the only selected therapy covered under the medical benefit due to its intravenous (IV) administration.

Three of the five therapies had evidence of GC reduction. Adalimumab, rilonacept, and sarilumab demonstrated significant reductions in GC use or GC elimination in their respective studies, while belimumab demonstrated non-significant GC dose reduction and eteplirsen had indirect GC reduction data available (**Table 1**).

Despite being priced at a substantial premium to low-cost GCs, all therapies evaluated were covered by some, if not all, of the 5 large national commercial plans with prior authorization using FDA indication or trial criteria as conditions for coverage (**Table 2**).

**Table 2:** Payer formulary and coverage policies of selected payer-covered GC-reducing therapies evaluated^a^.

Generic name (brand name)	Formulary status & PA criteria	National commercial health plans					Payer management summary
		Aetna	Humana	Anthem	United HealthCare	Cigna	
Adalimumab (Humira)	Formulary status	Covered					Covered by all payers generally at PA to trial criteria
	PA criteria	PA beyond FDA indication and trial criteria	PA to trial criteria			
Belimumab (Benlysta)	Formulary status	Covered				Non-formulary	Covered by 4 of 5 payers most commonly at PA to trial criteria
	PA criteria	PA to trial criteria		PA beyond FDA indication and trial criteria	PA to trial criteria	
Eteplirsen (Exondys 51)	Formulary status	Covered					Classified as a medical benefit product due to its IV formulation by most plans
	PA criteria	PA to trial criteria			PA beyond FDA indication and trial criteria	PA to trial criteria
Rilonacept (Arcalyst)	Formulary status	Non-formulary		Covered	Non-formulary	Covered	Managed at PA to FDA indication at n=2 plans; others required step therapy
	PA criteria	PA to trial criteria	PA to FDA indication		PA to trial criteria	
Sarilumab (Kevzara)	Formulary status	Covered		Non-formulary		Covered	Covered by most payers at PA to FDA indication
	PA criteria	PA to trial criteria	PA to FDA indication	PA to trial criteria	PA to FDA indication	

**Table 2** summarizes the formulary status and PA criteria for each of the 5 GC-reducing therapies in 5 national commercial health plans and provides an overall summary of payer management decisions for these selected therapies.

^a^Based on 2023 published formularies from the health plans listed in **Table 2**.

FDA, Food and Drug Administration; IV, intravenous; PA, prior authorization.

Pink boxes indicate non-formulary status. Green boxes indicate covered formulary status. Light green boxes indicate PA required.

##### Adalimumab

All plans currently cover adalimumab; similarly, all plans enforce trial criteria for adalimumab formulary coverage in RA, including failure, contraindication, and intolerance to a disease-modifying therapy.

##### Belimumab

Belimumab is covered on Aetna, Humana, Anthem, and United HealthCare and is non-formulary on Cigna. Plans enforce belimumab’s inclusion/exclusion criteria from its clinical trials.

##### Eteplirsen

All plans currently cover eteplirsen; all plans manage eteplirsen based on its trial inclusion/exclusion criteria, except United HealthCare, which is the only plan that adopted requirements beyond the trial criteria, as it also requires submission of a North Star Ambulatory Assessment or Gower’s test score.

##### Rilonacept

Rilonacept is non-formulary at Aetna, Humana, and United HealthCare. Aetna and United HealthCare manage rilonacept based on trial criteria, which specifies that patients either currently receive or demonstrate failure to one or more common therapies (including GCs). Humana manages rilonacept according to its labeled FDA indication, requiring a confirmed diagnosis of recurrent pericarditis (RP) demonstrated by symptoms and history of RP episodes. Rilonacept is covered by Anthem and Cigna; Anthem manages rilonacept by its FDA indication and Cigna manages rilonacept based on trial criteria.

##### Sarilumab

Sarilumab is covered on Aetna, Humana, and Cigna, while it is non-formulary at Anthem and United HealthCare. Most plans cover sarilumab following FDA indication criteria and require a demonstration of an inadequate patient response or intolerance to GCs.

### Cross-sectional study (qualitative interviews with payers)

#### Payer characteristics

A total of 14 US payers were screened; 13 payers were invited to complete the qualitative interviews. Among them, 4 were national MCO payers, 3 were regional MCO payers, 3 were PBM representatives, and 3 were managed Medicaid payers. The characteristics of payers who participated in the qualitative interviews are provided in **Supplementary Table 1**.

#### Summary of payer insights on the management of selected therapies

The summary of payer insights on coverage and formulary management of select GC-reducing therapies is presented in **Table 3**. When crafting coverage criteria and formulary structure, payers stated that they placed primary focus on each trial’s safety outcomes and primary endpoints. Although GC dose reduction data were meaningful to payers, it was not the primary driver in their decision-making due to the following reasons: none of the select GC-reducing therapies had GC reduction as a primary or secondary outcome, some GC reduction data were not statistically significant, trial design to assess GC reduction had too small of a sample for too short of a time period, and implications of contracting dynamics and rebates.

**Table 3:** Summary of payer management insights for selected GC-reducing therapies.

Generic name (brand name)	Current indication	GC reduction evidence summary	Summary of payer insights from interviews
Adalimumab (Humira)	RA^a^	Demonstrated significant reductions in oral/IV GC use, daily GC dose, and non-drug medical costs (*P*<0.01) (22, 23)	• Payers noted GC reduction minimally affects RA coverage amid a crowded market and contracting dynamics • Payers stated adalimumab was the preferred choice across multiple indications, driven by net rebates and portfolio contracts
Belimumab (Benlysta)	SLE^b^	Demonstrated non-significant reductions in GC dose across 3 pivotal phase 3 studies and in an RWE study (14–18)	• Payers stated GC reduction data influenced initial approval; its lack of statistical significance was not robust enough to remove the GC step therapy requirement • Some payers suggested that if belimumab demonstrated statistically significant GC reduction, they could favor belimumab over its competitor anifrolumab-fnia (Saphnelo)
Eteplirsen (Exondys 51)	DMD^c^	RWE analysis demonstrated that eteplirsen monotherapy resulted in significant slowing of respiratory decline vs. GCs alone (24, 25)	• Payers focused on eteplirsen’s lackluster efficacy outcomes given DMD’s associated morbidity, mortality, and severe complications • Payers noted that once competitors emerge, RWD in GC reduction can help its favorable coverage
Rilonacept (Arcalyst)	RP^d^	Demonstrated GC elimination into rilonacept monotherapy in pivotal Phase 3 trial; did not test for statistical significance (26, 27)	• Payers stated GC reduction had limited value in decision-making due to small patient numbers (N=14) and a short run-in period (12 weeks); primary and key secondary outcomes and safety were prioritized
Sarilumab (Kevzara)	PMR^e^	Demonstrated significant reductions in total actual cumulative prednisone-equivalent GC dose vs. placebo at week 52 (*P*<0.0001) (19, 20)	• Payers noted that GC reduction data in rare diseases that require lifelong GC use was valuable; however, payers prioritized the primary endpoint over GC reduction data in decision-making

**Table 3** presents the GC reduction evidence for each of the 5 GC-reducing therapies and summarizes qualitative payer insights obtained during this study.

^a^Reducing signs and symptoms, inducing major clinical response, inhibiting the progression of structural damage, and improving physical function in adult patients with moderately to severely active RA. ^b^Adult patients with active SLE who are receiving standard therapy. ^c^DMD in patients who have a confirmed mutation of the DMD gene that is amenable to exon 51 skipping. ^d^RP and reduction in risk of recurrence in adults and children ≥12 years. ^e^Adult patients with PMR who have had an inadequate response to corticosteroids or who cannot tolerate corticosteroid taper.

DMD, Duchenne muscular dystrophy; GC, glucocorticoid; PMR, polymyalgia rheumatica; RA, rheumatoid arthritis; RP, recurrent pericarditis; RWD, real-world data; RWE, real-world evidence.

#### Qualitative insights from payers on the impact of long-term GC use and the importance of GC reduction

All payers recognized the moderate clinical and economic burden associated with long-term GC use in chronic conditions due to their downstream complications (**Table 4**). When payers were asked to rate the clinical burden and economic burden associated with long-term maintenance treatment of GCs for the management of chronic conditions on a scale of 1 to 7 (with 1 being very low burden and 7 being very high burden), the mean (standard deviation [SD]) payer perception rating of the clinical burden was a 4.9 (1.0; n=12) out of 7, while the perception of the economic burden was rated as a 3.5 (1.0; n=13) out of 7. All payers understood the clinical burden of GCs, noting that GC side effects were a significant contributor to the clinical burden in patients, requiring the need to use the lowest possible dose or replace GCs with other therapies. Although the economic consequences are not well understood by payers, they stated that the side effects of GCs can also carry an economic burden for payers.

**Table 4:** Qualitative insights from interviews with payers.

Theme	Quotes from interviews with payers
Payers understand the clinical burden of GCs, rating it a 4.5 out of 7. However, economic consequences are not well understood and were rated 3.5 out of 7.	“I’d give [the clinical burden rating] a 5 [out of 7]. Everyone knows the long-term chronic, use of steroids and its effect on the body, and how we try to take vacation breaks by using the lowest dose, combined with/replaced by current drugs.” “I will give economic burden a 5 [out of 7]. The bone mass density, edema, developmental disorders, and all of that can be the issues. Its safety based.” “Economic consequences are not well understood. The development of diabetes is a serious consequence, though. Not everyone is a high-dose user.”
When evaluating clinical trial data, payers focus primarily on primary endpoints. GC reduction data were not a primary driver of decision-making across the 5 analogs because they were all secondary or exploratory endpoints.	“[GC reduction] is part of our 5 criteria since we look at secondary endpoints. It’s usually not a primary endpoint, which we look at with more importance; but it is part of the compelling evidence we see. I don’t think I’ve made a P&T [Pharmacy & Therapeutics] decision solely based on the fact that a drug is steroid-reducing. It is a data point that is impactful to our coverage decision, but usually isn’t the main decider.” “[Management] is based on primary endpoint data, population size, disease state, available treatments, FDA labeling, potential for inappropriate use, and contracting—not whether it’s steroid-reducing or not.”
In chronic rare diseases, GC reduction is an acceptable primary endpoint. For sarilumab, the only analog in a rare indication that required lifelong GCs, GC reduction was important for coverage.	“I would see steroid-reducing as acceptable as a primary endpoint [for a new therapy in a rare, chronic disease state]. If they are able to maintain the same level of disease control as they did with steroids. If it were a condition where long-term baseline steroids cause growth defects, like in a pediatric population, I would accept it as a primary. I don’t really care if it is primary or secondary.” “There is a lot more concern around pediatrics, so my rating on its impact on coverage would jump to a 5 [out of 7], and steroid reduction must be in association with efficacy.” “[For sarilumab, GC reduction] was important here for coverage. It was the first to market. It did help us with the pricing. It helped justify the price and not put strict criteria on dosing or stepping. If it did not reduce steroids, I’m not sure we would cover it. PMR requires steroids for a long period of time. It is a requirement in a disease like this to reduce steroid use. Without steroid reduction, we would not have covered [it]. If this condition had other drugs available, it would be different.”
It is important to remind payers of the QoL and long-term impacts of GCs.	“It is always good to remind payers that quality of life, downstream diseases, and lifelong growth defects are all affected by steroids. It would be helpful to have payer contextualization of how much steroids would actually make a difference.”

**Table 4** describes the overall themes obtained from discussions with payers during this study and the key verbatim quotes that support the themes.

FDA, Food and Drug Administration; GC, glucocorticoid; PMR, polymyalgia rheumatica; QoL, quality of life.

Payers were asked about the extent to which GC reduction data impacted their decision-making about each of the 5 therapies and indications presented (**Table 4**). While GC reduction impacted payer decision-making to some degree for all 5 therapies, GC reduction data were noted as having the largest impact on sarilumab. Indeed, sarilumab’s indication in a rare disease, PMR, and the available GC reduction data that demonstrated the impact of long-term GC use were factors to this.

In contrast, payers noted that GC reduction had the lowest impact on their decision-making regarding eteplirsen, which was due to concerns about eteplirsen’s lack of efficacy in DMD and poor safety outcomes. Payers also noted that rilonacept and adalimumab demonstrating GC reduction in their respective trials was a secondary consideration in their coverage decisions, with the primary efficacy and safety endpoints being the primary consideration. Notably, regarding therapies with multiple competitors in their therapeutic area, such as adalimumab, payers were most concerned with pricing and contracting. Nonetheless, payers stated they had an interest in future studies that include GC reduction as a primary endpoint, especially if the therapy maintained the same level of disease control as GCs, and thought robust GC reduction data were particularly relevant in cases of rare, chronic diseases without specific alternative treatment options and in pediatric populations. Lastly, payers agreed education on the value of GC reduction would increase their understanding of the clinical and economic impact of GCs.

#### Other considerations of payers regarding therapy management

Throughout the survey, payers were asked to consider and discuss the expected impact on therapy management of label indication, efficacy, and safety; disease education; messaging; cost offsets; and guidelines/key opinion leader input. Payers thought that label indication, efficacy, and safety would have a very high impact on therapy management due to viewing FDA labeling as the most influential driver of coverage and management and the potential to include trial inclusion and exclusion criteria depending on competitor dynamics and budget impact. Payers also expected disease education to have a very high impact on therapy management, noting that it is crucial in payer coverage and management outcomes and understanding the clinical burden of long-term GC use. Next, they noted the importance of first-to-market therapies in diseases with high unmet need and cost offsets allowing for preferential tiering of therapies. Lastly, payers rated guidelines/key opinion leader input as having a neutral impact on management, citing that guidelines rarely trigger a Pharmacy & Therapeutic Committee re-review and subsequent policy changes unless there are major changes in the efficacy and/or safety of a therapy.

## Discussion

To our knowledge, this is the first publication that characterizes US payer perspectives regarding the clinical burden of GCs when making formulary decisions on GC-reducing medications. Our qualitative interviews with payers revealed that reduction of negative clinical outcomes accompanying long-term GC use was clinically important when making their coverage decisions on the 5 selected GC-reducing therapies. Despite carrying high costs, all selected medications were covered in most national commercial health plans in place of, or in addition to, GCs.

The 5 selected GC-reducing therapies were purposefully diverse, and each had unique characteristics that made payer decision-making multi-factorial and complex, including factors such as pricing and contracting agreements/dynamics. Importantly, none of the selected therapies included GC reduction as a primary endpoint in their respective clinical trials. Given that payers reported they evaluate new therapies based on their primary endpoints and had never evaluated a therapy with GC reduction as a primary endpoint, they were interested in seeing future studies with GC reduction as a primary endpoint.

Other factors that may contribute to payer decision-making included safety and real-world evidence. Payers reported in this survey that safety has a high impact on therapy management. Although this study did not specifically review safety data or real-world evidence, these factors could influence payer attitudes. When GC reduction data serves as a primary or secondary endpoint in a trial, especially in a population with high unmet need (e.g., in a rare condition, a pediatric population, or in a disease area with limited therapeutic alternatives), GC reduction may serve as a key driver in payer decision-making.

Ultimately, payer decisions on medication coverage and access can impact a clinician’s ability to optimize patient care. The medications reviewed in this study were covered under most of the insurance policies but their use often required criteria that had to be met, or a trial of step therapy. While uncertainty in access or coverage may delay treatment or potentially allow further disease progression, clinicians can advocate for coverage of essential therapies that have clinical value to improve patient outcomes. Payers in this study reported trials with GC reduction as a primary endpoint would be particularly important in cases of rare, chronic diseases without specific alternative treatment options and in pediatric populations, highlighting their priorities and identifying a path for clinicians and payers to align on the clinical relevance and access to medications in these rare, persistent diseases where chronic use of GCs has shown numerous adverse effects on patients (28–33).

One such rare disease is CAH, which impacts pediatric and adult populations. Under a GC-only treatment paradigm for CAH, to restore the HPA axis negative feedback loop and reduce excess ACTH and adrenal androgen production, supraphysiologic doses of GCs are typically required and may be associated with additional risks and side effects including decreased growth rates, impaired final adult height, and reduced bone health (28, 29, 34–37). Risk of bone fractures, cardiovascular disease, and mental health problems due to exposure to GCs, as well as adrenal androgens, are also common in CAH (38–40). The negative clinical outcomes of chronic GC use highlight an urgent need for clinicians to address their adverse effects, minimize long-term usage and dose, and find safe and effective therapeutic alternatives. Striking the appropriate balance between clinical need and access to medications is crucial. While it can be challenging for clinicians when GC-reducing therapies are not adequately covered by health plans in the US, healthcare providers often have the ability to influence payer coverage by substantiating the clinical need for the medications they prescribe. In rare conditions where payers understand the value of GC reduction, new therapeutic options can offer value to clinicians, payers, and patients by mitigating long-term safety concerns while maintaining disease control.

## Limitations

The limitations of this research should be considered alongside the results. First, due to the study’s cross-sectional research design, recall or social desirability bias may be present. However, the questions in the web-enabled screener and qualitative interviews were framed to help mitigate these biases and payers were asked to provide responses to the best of their ability. Additionally, this study recruited a relatively small sample, reducing its generalizability. However, the sample was well distributed between a variety of organizations (e.g., national and regional MCOs, PBM representatives, and Medicaid). Also, some additional factors may contribute to coverage decision-making including the drug’s entire safety profile, long-term real-world data, adherence, or perspectives from patients and clinicians. Providing an individual risk-benefit analysis of each identified GC-reducing therapy would have provided respondents with additional perspective to consider but was out of scope for the current study based on time restrictions. The 5 GC-reducing therapies were identified based on prespecified criteria and were used to proxy how payers may consider a GC-reducing therapy for CAH. However, applications of GC-reducing therapies vary by disease state and potential for disease modification, which may impact payer coverage decisions, and thus, cannot be completely proxied or captured and limit generalizability. Lastly, the opinions expressed by the payers in the qualitative interviews do not necessarily reflect the opinions or data findings of the sponsor or all payers.

## Conclusions

Our research shows that each GC-reducing therapy evaluated was covered in place of, or in addition to, GCs despite carrying a premium price. While GC reduction was meaningful to payers, it was a secondary driver in coverage decision-making due to contracting dynamics, lack of competitors, small sample size in trials, and most importantly, payers’ focus on the trials’ primary endpoints. Payers acknowledged the clinical and economic value of reducing long-term GC use and may consider GC reduction data in their decision-making. Future studies with GC reduction as a primary endpoint in rare and chronic diseases that affect pediatric populations may inform access decision-making. Clinicians who understand how payers evaluate GC-reducing therapies and payer priorities can help shape more patient-centered coverage decisions and better align treatment decisions to ensure optimal patient management.

## Author's note

Previously presented as poster at AMCP Nexus 2024, Las Vegas, NV, Oct 14-17.

## Data Availability

The raw data supporting the conclusions of this article will be made available by the authors, without undue reservation.
