# Supplementary material for: Optimizing glucocorticoid therapy in congenital adrenal hyperplasia and analog conditions: the intersection of dose reduction, patient care, and coverage in the US
Source: Front Endocrinol (Lausanne). 2025 Sep 9;16:1603701. doi: 10.3389/fendo.2025.1603701 (PMC12454079; doi:10.3389/fendo.2025.1603701)
Supplement: Supplementary file 1 [file Table1.docx]

**Supplementary Materials**

Supplementary Table 1. Payer characteristics: Primary research

| **Payer characteristics** |  |
| --- | --- |
| **Organization type, n** | **13** |
| National MCO, n (%) | 4 (30.8) |
| Regional MCO, n (%) | 3 (23.1) |
| PBM, n (%) | 3 (23.1) |
| Medicaid, n (%) | 3 (23.1) |
| **US covered lives, n** | **141,100,000** |
| Commercial, n (%)^a^ | 102,400,000 (72.6) |
| Medicaid, n (%)^a^ | 24,000,000 (17.0) |
| Medicare, n (%)^a^ | 14,700,000 (10.4) |
| **US commercial-covered lives, n** | **102,400,000** |
| National MCO, n (%)^b^ | 69,100,000 (67.4) |
| PBM, n (%)^b^ | 30,500,000 (29.7) |
| Regional MCO, n (%)^b^ | 2,900,000 (2.8) |

^a^ Percentages calculated with the denominator as the total US covered lives.

^b^ Percentages calculated with the denominator as the total US commercial-covered lives.

MCO=managed care organization; PBM=pharmacy benefit manager; US=United States.
